# Xinnao Shutong Modulates the Neuronal Plasticity Through Regulation of Microglia/Macrophage Polarization Following Chronic Cerebral Hypoperfusion in Rats

**DOI:** 10.3389/fphys.2018.00529

**Published:** 2018-05-15

**Authors:** Liye Wang, Rongliang Wang, Zhigang Chen, Haiping Zhao, Yumin Luo

**Affiliations:** ^1^Cerebrovascular Diseases Research Institute, Xuanwu Hospital, Capital Medical University, Beijing, China; ^2^Dongfang Hospital, Beijing University of Chinese Medicine, Beijing, China

**Keywords:** chronic cerebral hypoperfusion, neuronal plasticity, cognitive impairment, microglial polarization, Xinnao shutong

## Abstract

Xinnao shutong (XNST) capsules have been clinically used in China to treat cerebrovascular diseases. Previous studies have demonstrated that XNST has significant neuroprotective effects against acute cerebral ischemic stroke. The present study investigated the effects and mechanisms of XNST treatment following chronic cerebral hypoperfusion. Thirty-six adult male Sprague-Dawley rats were treated with XNST or vehicle following permanent bilateral common carotid artery (BCCA) ligation. Body weight was recorded on days 0, 3, 7, 14, 28, and 42 post-surgery. The Morris water maze (MWM) test was used to assess cognitive function in rats. Immunofluorescent staining and western blot were used to assess the severity of neuronal plasticity, white matter injury, and the numbers and/or phenotypic changes incurred to microglia. Protein levels of p-AKT (Thr308) and p-ERK (Thr202/Tyr204) were detected 42 days after BCCA ligation was performed. The results indicate that XNST treatment significantly reduced escape latency, decreased the frequency of platform crossing compared to the vehicle group. Synaptophysin, protein levels improved and white matter injury ameliorated following XNST treatment. Meanwhile, XNST reduced the number of M1 microglia and increased the number of M2 microglia. Furthermore, p-AKT (Thr308) and p-ERK (Thr202/Tyr204) levels were increased 42 days following BCCA ligation. In summary, our results suggest that XNST mitigates memory impairments by restoration of neuronal plasticity and by modulation of microglial polarization following chronic cerebral hypoperfusion in rats.

## Introduction

Cerebral circulation disturbances cause numerous neurological and psychiatric illnesses (e.g., epilepsy), and are also associated with a decline in cognitive function (e.g., Alzheimer’s disease). It is important to understand the effects of cerebral hypoperfusion-related pathological changes on cognitive dysfunction, and to explore potential targets for effective therapies. Recent studies have highlighted that neuronal plasticity is closely related to depression and memory (Castren and Hen, 2013; Busceti et al., 2015). Microglia contribute to neuronal plasticity through modulates the synaptogenesis and neuronal maturation in the healthy brain (Delpech et al., 2015). However, any disruption of microglial function can result in neuronal plasticity and cognitive function impaired (Sominsky et al., 2018). In addition, white matter damage is an important cause of cognitive deficits and is often associated with microglial activation (Choi et al., 2016).

Microglia/macrophages are the primary mediators of the immune system in the central nervous system (CNS), and are integral to subsequent inflammatory responses (Loane and Byrnes, 2010; Venkatesan et al., 2010). Microglia/macrophages can assume diverse phenotypes, including the two polarized phenotypes, M1 and M2, and play different roles in specific conditions. The pro-inflammatory M1 phenotype favors the production and release of cytokines that exacerbate neuronal injury (Hu et al., 2012; Boche et al., 2013). In contrast, the M2 phenotype favors the release of neurotrophic factors that promote the repair and regeneration of the injured nerve (Hu et al., 2012, 2015). Interestingly, previous studies have confirmed that the phenotypic changes observed in microglia/macrophages are associated with the AKT and ERK signaling pathways (Wang et al., 2015, 2016).

Xinnao shutong (XNST) capsules, produced in China, were provided by Jilin Aodong Taonan Pharmaceutical Co., Ltd. (National Medical Number: Z22021965). Previous studies have demonstrated that XNST mediates neuroprotective effects by inhibiting apoptosis and by inducing angiogenesis during acute cerebral ischemia (Zhang et al., 2008; Qian-Song et al., 2015). However, whether XNST has neuroprotective effects on chronic cerebral hypoperfusion remains unknown. In this study, we used a rat model of permanent bilateral common carotid artery (BCCA) ligation to analyze the effects of XNST on neuronal plasticity and microglia/macrophage polarization following chronic cerebral hypoperfusion.

## Materials and Methods

### Animals

Thirty-six male Sprague-Dawley rats (280–300 g) were purchased from Vital River Laboratory Animal Technology Co., Ltd. (Beijing, China). Five rats were kept in a cage and allowed free access to water and food. The rodent house was maintained at 22 ± 2°C with a 12 h/12 h light/dark cycle. The study was approved by the Institutional Animal Care and Use Committee of the Capital Medical University, and was in accordance with the principles outlined in the National Institutes of Health’s Guide for the Care and Use of Laboratory Animals.

### BCCA Surgery

Bilateral common carotid artery surgery was performed on the rats as previously described (Cho et al., 2017). Briefly, rats were initially anesthetized using 5% isoflurane in 70% nitrogen and 30% oxygen. Anesthesia was maintained during the surgical procedure with 3% isoflurane, using a face mask. Through a midline incision, the BCCA were exposed and freed from their sheaths. Two 4–0 silk sutures were placed around the distal and proximal parts of the right CCA. The same surgical procedure was performed on the left CCA 30 min later. The sham group received the same surgical operation without ligation of the carotid arteries. Rectal temperature was maintained at 36.5–37.5°C with the use of a heat pad throughout the surgical procedure. The animals were then allowed to recover from anesthesia and were returned to their cages.

### Grouping and Treatment

**FIGURE 1:**
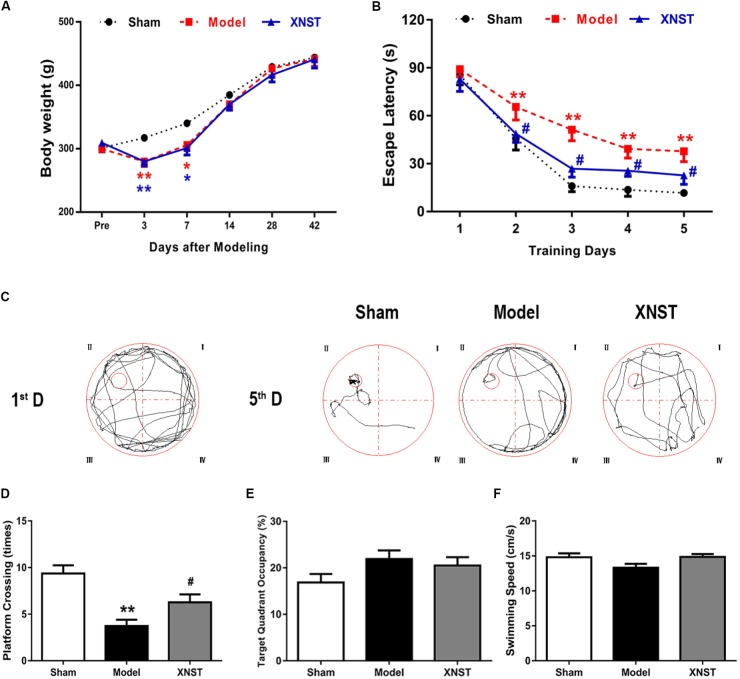
Xinnao shutong (XNST) ameliorates cognitive function following chronic cerebral hypoperfusion. **(A)** Comparisons of weight for each group on day 0, 3, 7, 14, 28, and 42. **(B)** The escape latency of rats in the training trials of the hidden platform task. **(C)** Representative pathways for the first and last training day of the hidden platform task for each group. **(D)** Frequency of platform crossing in the probe trial. **(E)** Percentage of time spent in the target quadrant in the probe trial. **(F)** Swimming speed in the probe trial. Values are expressed as mean ± SEM (*n* = 12). ^∗^*p* < 0.05 vs. Sham, ^∗∗^*p* < 0.01 vs. Sham, ^#^*p* < 0.05 vs. Model, ^##^*p* < 0.01 vs. Model.

The Sprague-Dawley rats were randomly divided into three groups: Sham group, Model group, and XNST group (*n* = 12). Vehicle and drug treatment was initiated on day 1 following BCCA ligation or sham surgery, and was continuously administered intragastrically until day 42. Doses were calculated according to body surface area, and were based on human clinical doses. The experimental design is illustrated in **Figure 1**. All rats were weighed on days 0, 3, 7, 14, 28, and 42 post-surgery. Six rats from each group were chosen for western blot analysis, and the six other rats were chosen for immunofluorescent staining.

### Morris Water Maze Test

Cognitive function was examined using the Morris water maze (MWM) test, as previously described, from days 36–42 post-BCCA ligation (Wang et al., 2013a; Qian-Song et al., 2015). The maze consisted of a black circular pool (120 cm diameter, 40 cm height) and a 10 cm diameter plexiglas platform. In this test, the pool was filled with water (22 ± 1°C) to a depth of 20 cm, and the platform was submerged 1 cm under the water’s surface in order to be hidden from the rat’s view. The test consisted of two phases: (1) acquisition trial, and (2) probe trial. For the acquisition trial phase, rats underwent four trials per day for five consecutive days to locate the hidden platform in 120 s. The escape latency(s) was recorded. At the end of each trial, the rats were gently guided to the platform and allowed to remain on it for an additional 20 s. The probe trial was conducted on day 6. For this, the platform was removed and each rat was placed in the pool one time for 120 s, with the starting location situated farthest from the platform. The time spent in the target quadrant, the frequency of platform crossing, and the swim speed was recorded. Typical swimming pathways on the first and last day of training for the hidden platform task was recorded.

### Western Blot Analysis

Rat brains were weighed and homogenized in lysis buffer, which contained 50 mmol/L Tris-HCl, pH 7.5, 100 mmol/L NaCl, 1% Triton X-100 (RIPA, CST, United States) and protease inhibitors (Cocktail, Sigma-Aldrich, St. Louis, MO, United States). Equal amounts of protein (25 μg) were separated with sodium dodecylsulfate-polyacrylamide gel electrophoresis (SDS–PAGE), followed by electrophoretic transfer to polyvinylidene difluoride membranes. Membranes were incubated overnight at 4°C with a 1:1000 dilution of primary antibodies against MAP2 (Cell Signaling Technology, Danvers, MA, United States), synaptophysin (Abcam, Cambridge, MA, United States), Iba1 (Abcam, Cambridge, MA, United States), iNOS (Abcam, Cambridge, MA, United States), CD16 (Abcam, Cambridge, MA, United States), Arg1 (Cell Signaling Technology, Danvers, MA, United States), CD206 (Abcam, Cambridge, MA, United States), p-AKT (Thr 308) (Cell Signaling Technology, Danvers, MA, United States), AKT (Cell Signaling Technology, Danvers, MA, United States), p-ERK (Cell Signaling Technology, Danvers, MA, United States), and ERK (Cell Signaling Technology, Danvers, MA, United States). The chemiluminescent detection of antigens was performed with horseradish peroxidase-conjugated secondary antibodies (Santa Cruz, CA, United States) for one hour at room temperature. Immunoblots were probed using an ECL plus chemiluminescence reagent kit (Millipore, MA, United States) and then visualized with a computerized image analysis system (Fluro Chen 2.0, Olympus, Japan). The integrated density values were normalized to β-actin, and were calculated using ImageJ software (Rawak Software, Inc., Germany).

### Immunofluorescent Staining

Rats were euthanized 42 days after BCCA ligation with intraperitoneal injections of chloral hydrate (300 mg/kg) and perfusion with cold saline. Brains were subsequently placed in phosphate-buffered saline (PBS) and 4% formaldehyde in PBS and were dehydrated in a 30% sucrose solution in PBS for 48 h. Following cryoprotection, frozen brains were sectioned coronally into 20 μm thick slices and were then subjected to immunofluorescent staining. Primary antibodies included MAP2 (Cell Signaling Technology, Danvers, MA, United States), synaptophysin (Abcam, Cambridge, MA, United States), NeuN (Millipore, Burlington, MA, United States), MBP (Abcam, Cambridge, MA, United States), CD16 (BD Pharmingen, San Diego, CA, United States), Arg1 (Cell Signaling Technology, Danvers, MA, United States) and Iba1 (Wako Pure Chemical Industries, Osaka, Japan). Following incubation for 2 h in a blocking solution, containing 3% normal donkey serum and 0.3% Triton X-100 in PBS, sections were incubated in primary antibodies. Subsequently, sections were incubated in a mixture of fluorescent secondary antibodies (Alexa 488/Alexa 594-conjugated anti-mouse/anti-rabbit IgG). The sections were counterstained with 4′,6-diamidino-2-phenylindole (DAPI). All images were acquired using a fluorescence microscope (Carl Zeiss, Germany). The intensity value of MAP2 and synaptophysin staining in the region of cortex, hippocampal CA1, CA3, the intensity value of MBP staining in the region of cortex, striatum and corpus callosum was calculated using the ImageJ (National Institutes of Health, Bethesda, MD, United States).

### Statistical Analysis

Data analyses were performed using SPSS v11.0 (SPSS Inc., Chicago, IL, United States). The data from the hidden platform trials were analyzed with two-way analysis of variance. For this, mean escape latency was the dependent variable, day was the within-subject variable, and the three groups were the between-subject variable. Where appropriate, *post hoc* comparisons were assessed using the Least Significant Difference test (equal variances assumed) or Dunnett’s T3 test (equal variances not assumed). One-way analysis of variance was performed to the remaining data, and was performed to determine the significance of the effects of XNST treatment in BCCA rats. Unless otherwise specified, *P* < 0.05 was considered significant. All data are expressed as the mean ± SEM.

## Results

### XNST Ameliorates Cognitive Function Following Chronic Cerebral Hypoperfusion

In the first week following surgical intervention, a decrease in body weight was observed in both the Model and XNST groups (**Figure 1A**). The body weight of the Model and XNST groups were significantly lower than that of the Sham group (**Figure 1A**, *P* < 0.05). At days 14, 28, and 42 post BCCA ligation, there were no significant differences across the three groups.

Cognitive impairment caused by BCCA ligation was assessed using the MWM test. The escape latency(s), to find the hidden platform, during the acquisition session in all rats are displayed in **Figure 1B**. During training, rats in the Model group exhibited the longest escape latencies. Comparisons of individual day values are as follows: Escape latency gradually decreased over time across all groups. The Model group rats began to have notably longer escape latencies compared to the Sham rats from the second day of training (**Figure 1B**, *P* < 0.05). This difference was significant for 4 days. Furthermore, from the second day to the last day, the XNST group had significantly shorter escape latencies than the Model group (*P* < 0.05).

When examining the typical swimming pathways in each group for the first and last day of training for the hidden platform task, the results indicate that the rats in the Model group used an inappropriate searching strategy to locate the hidden platform, which resulted in longer latency periods (**Figure 1C**). The results indicate that BCCA ligation significantly decreases the frequency of platform crossing. However, compared to the Model group, the XNST group increased the frequency of platform crossing (**Figure 1D**, *P* < 0.05). Furthermore, the percentage of time spent in the target quadrant was used to evaluate performance retention, and no significant differences were observed across the three groups (**Figure 1E**). To rule out any interference of sport ability in the study, we evaluated swimming velocity in the rats. No significant differences were observed in swimming velocity across the three groups (**Figure 1F**).

### XNST Regulates Neuronal Plasticity Following Chronic Cerebral Hypoperfusion

**FIGURE 2:**
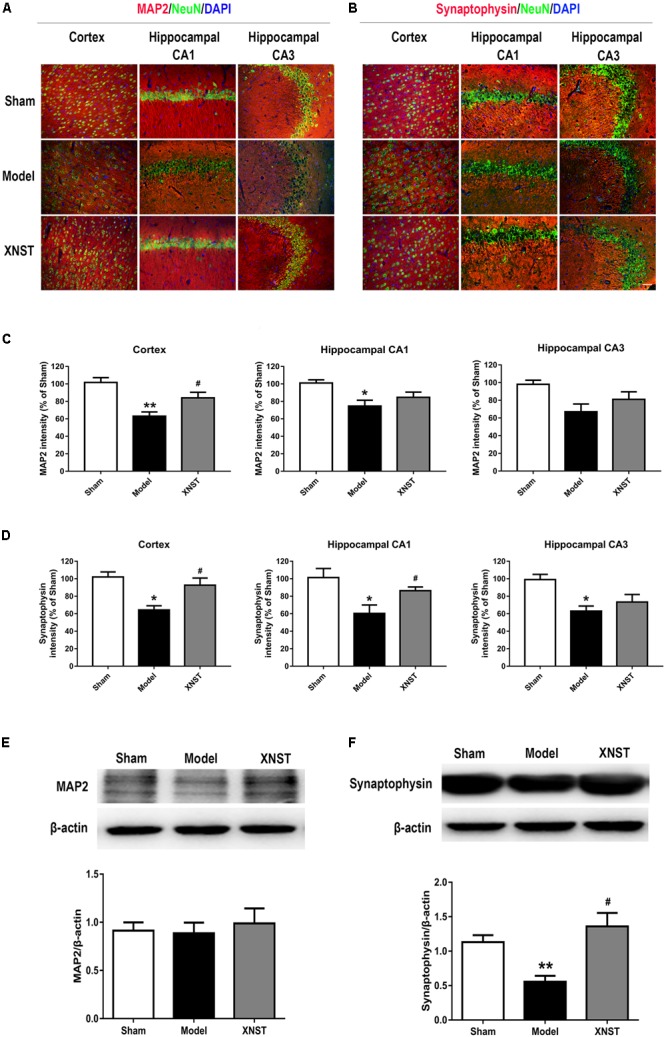
Xinnao shutong regulates neuronal plasticity following chronic cerebral hypoperfusion. **(A)** Representative immunofluorescence images showing colocalization of MAP2 (red) and NeuN (green) in cortex, hippocampal CA1 and CA3 42 days following bilateral common carotid artery (BCCA) ligation. DAPI (blue) indicates cell nuclei. **(B)** Representative immunofluorescence images showing colocalization of synaptophysin (red) and NeuN (green) in cortex, hippocampal CA1 andCA3. DAPI (blue) indicates cell nuclei. **(C,D)** Quantification of the relative MAP2 and synaptophysin immunostaining intensity in different regions. **(E,F)** Western blot detection and quantitative analysis of MAP2 and synaptophysin. Scale bar = 50 μm. Values are expressed as mean ± SEM (*n* = 12). ^∗^*p* < 0.05 vs Sham, ^∗∗^*p* < 0.01 vs Sham, ^#^*p* < 0.05 vs Model.

To investigate neuronal plasticity in rats who underwent BCCA ligation and the protective effects of XNST, we measured protein levels of MAP2 and synaptophysin using immunofluorescent staining and western blot analysis. Results from the immunofluorescent staining experiments showed poor NeuN and MAP2 staining in the Model group, whilst staining for NeuN and MAP2 was significantly improved in the XNST group (**Figure 2A**). Additionally, BCCA induced disordered staining for synaptophysin in the cortex and hippocampal regions (CA1 and CA3), which was attenuated by XNST treatment (**Figure 2B**). We also quantified the fluorescence intensity of the MAP2 and synaptophysin in different regions. The results showed that, compared to the Model group, the XNST treatment increased the MAP2 fluorescence intensity in cortex (**Figure 2C**, *P* < 0.05) and the synaptophysin fluorescence intensity in cortex, hippocampal CA1 (**Figure 2D**, *P* < 0.05) 42 days following BCCA ligation.

The western blot analysis showed that there was no significant difference in MAP2 levels across the three groups (**Figure 2E**). However, synaptophysin protein levels were significantly decreased in the Model group compared to the Sham group (**Figure 2F**, *P* < 0.01), and XNST significantly increased synaptophysin levels compared to the Model group (**Figure 2F**, *P* < 0.05).

### XNST Promotes White Matter Integrity Following Chronic Cerebral Hypoperfusion

**FIGURE 3:**
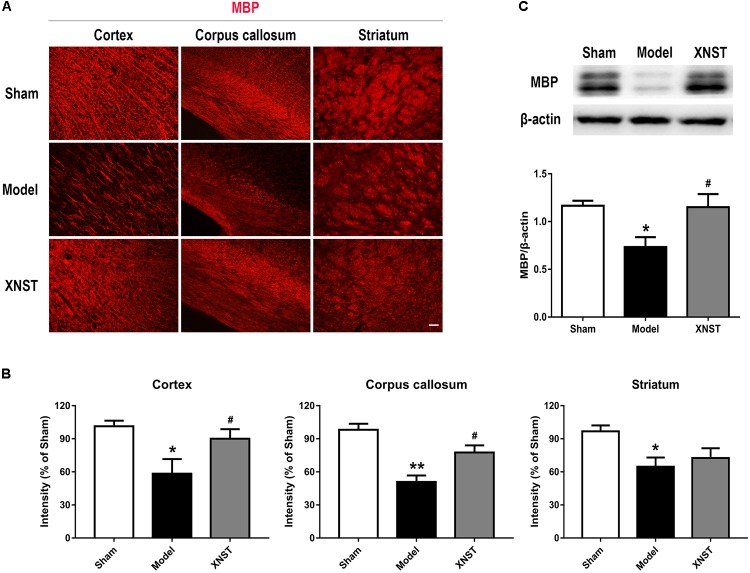
Xinnao shutong reduces white matter injury following chronic cerebral hypoperfusion. **(A)** Representative immunofluorescence images showing MBP in cortex, corpus callosum and striatum. Scale bar = 50 μm. **(B)** Quantification of the relative MBP immunostaining intensity in different regions. **(C)** Western blot detection and quantitative analysis of MBP. Values are expressed as mean ± SEM (*n* = 12). ^∗^*p* < 0.05 vs Sham, ^∗∗^*p* < 0.01 vs Sham, ^#^*p* < 0.05 vs Model.

We used immunofluorescent staining to evaluate damage incurred to the axons and myelin sheath in cortex, corpus callosum and striatum. This was achieved by assessing the myelin-associated proteins (MBP, a marker of myelination) to measure the loss of myelin (**Figure 3A**). At 42 day following BCCA ligation, MBP immunoreactivity was weak in the cortex, corpus callosum and striatum, reflecting demyelination of axons. XNST treatment significantly increased the MBP intensity in cortex and corpus callosum (**Figure 3B**, *P* < 0.05), suggesting the long-term preservation of demyelinated axons. Consistent with the immunostaining, the western blot showed that, compared to the Model group, the protein level of MBP was markedly reserved at day 42 following XNST treatment (**Figure 3C**, *P* < 0.05).

### XNST Attenuates Numbers of Microglia in Hippocampal CA1 and Dentate Gyrus Following Chronic Cerebral Hypoperfusion

**FIGURE 4:**
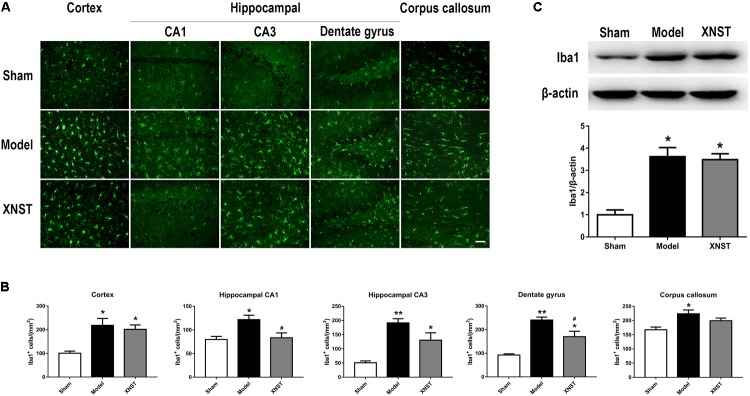
Xinnao shutong attenuates numbers of microglia in hippocampal CA1 and dentate gyrus following chronic cerebral hypoperfusion. **(A)** Representative immunofluorescence images showing Iba1-positive (microglial marker) cells in cortex, hippocampal CA1, CA3, dentate gyrus, and corpus callosum 42 days following BCCA ligation. Scale bar = 50 μm. **(B)** Quantitative analysis of Iba1^+^ cells in different regions. **(C)** Western blot detection and quantitative analysis of Iba1. Values are expressed as mean ± SEM (*n* = 12). ^∗^*p* < 0.05 vs. Sham, ^∗∗^*p* < 0.01 vs. Sham, ^#^*p* < 0.05 vs. Model.

Microglia are resident immune cells within the CNS that play a central role in the initiation and propagation of inflammatory responses. Immunofluorescent staining indicated that the numbers of Iba1-positive (a microglial marker) cells increased dramatically following BCCA ligation in the cortex, hippocampal CA1, CA3, dentate gyrus and corpus callosum. However, XNST inhibited the over-activation of microglia, as microglia density was reduced when compared to the Model group in hippocampal CA1 and dentate gyrus regions (**Figures 4A,B**, *P* < 0.05). Western blot analysis revealed that the protein levels of Iba1 were significantly increased in the Model and XNST groups compared to the Sham group (**Figure 4C**, *P* < 0.05). The mean Ibal protein level in the XNST group was lower, however, there was no significant difference in Iba1 levels between the Model and XNST groups.

### XNST Drives M2 Microglia/Macrophage Polarization Following Chronic Cerebral Hypoperfusion

**FIGURE 5:**
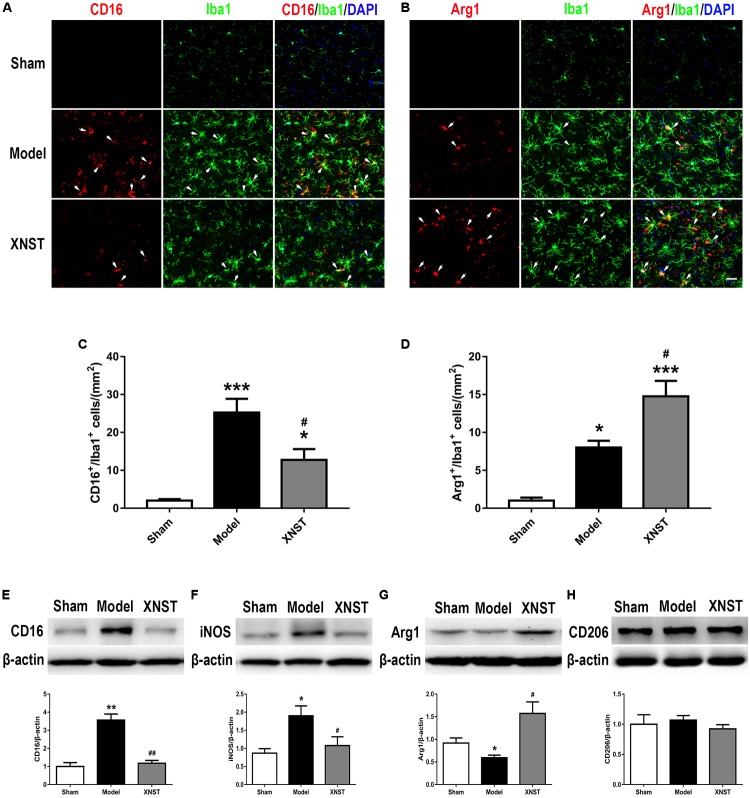
Xinnao shutong drives M2 microglia/macrophage polarization following chronic cerebral hypoperfusion. **(A,B)** Double immunofluorescent staining for M1 marker (CD16) or M2 marker (Arg1) (red) with microglial marker (Iba1) (green) in different groups at 42 day following BCCA ligation. **(C,D)** Quantification of microglia immunolabeled for CD16^+^/Iba1 and Arg1^+^/Iba1 cells. DAPI (blue) indicates cell nuclei. Scale bar = 25 μm. **(E–H)** Western blot detection and quantitative analysis of CD16, iNOS, Arg1, and CD206. Values are expressed as mean ± SEM (*n* = 12). ^∗^*p* < 0.05 vs. Sham, ^∗∗^*p* < 0.01 vs. Sham, ^∗∗∗^*p* < 0.001 vs. Sham, ^#^*p* < 0.05 vs. Model, ^##^*p* < 0.01 vs. Model.

Remarkably, microglia can play opposing roles in the face of cerebral injury. Polarized microglia/macrophages can be distinguished by the expression of different cell surface markers. To evaluate the polarization states of microglia/macrophages following chronic cerebral hypoperfusion-induced injury across the different groups, we further examined the expression of M1 (CD16) and M2 (Arg1) markers in Iba1^+^ microglia by double immunofluorescent staining (**Figures 5A,B**). The number of CD16- positive and Arg1-positive microglia in Model group were much higher than in sham group (**Figures 5C,D**, *P* < 0.05). Notably, compared to the Model group, the XNST treatment decreased the number of M1 microglia (CD16^+^/Iba1^+^ cells), while increased the number of M2 microglia (Arg1^+^/Iba1^+^ cells) (**Figures 5C,D**, *P* < 0.05).

To verity the immunofluorescent staining results, we detected the microglial markers by western blot. The results indicated that, the M1 markers CD16 and iNOS were significantly increased in the Model group. XNST reduced the expression of CD16 and iNOS compared to the Model group (**Figures 5E,F**, CD16, *P* < 0.01; iNOS, *P* < 0.05). The M2 markers, Arg1 and CD206 were also evaluated with western blot analysis. BCCA ligation decreased the expression of Arg1, and XNST treatment enhanced the levels of Arg1 (**Figure 5G**, *P* < 0.05). However, no significant differences were observed in CD206 levels between the Model and XNST groups (**Figure 5H**) at day 42 after BCCA ligation. These findings suggest that XNST promotes microglia polarization to the M2 phenotype.

### XNST Activates the AKT and ERK Signaling Pathways Following Chronic Cerebral Hypoperfusion

**FIGURE 6:**
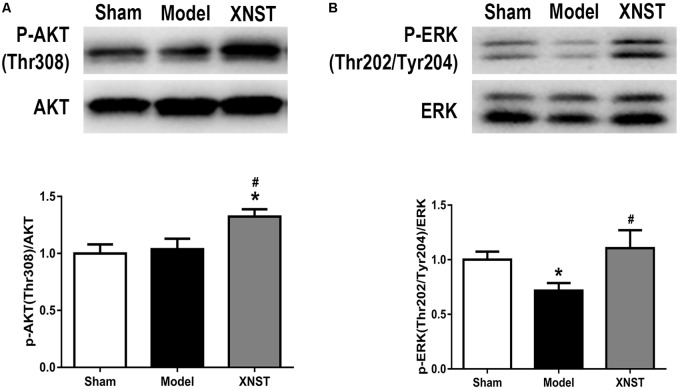
Xinnao shutong activates AKT and ERK signaling pathways following chronic cerebral hypoperfusion. **(A)** Western blot detection and quantitative analysis of p-AKT (Thr308). **(B)** Western blot detection and quantitative analysis of p-ERK (Thr202/Tyr204). Values are expressed as mean ± SEM (*n* = 12). ^∗^*p* < 0.05 vs. Sham, ^#^*p* < 0.05 vs. Model.

Previous studies have indicated that the AKT and ERK signaling pathways are microglia/macrophage polarization check points. In our study, the western blot analysis showed that XNST increased p-AKT (Thr308) and p-ERK (Thr202/Tyr204) protein levels compared to the Model group 42 days after chronic cerebral hypoperfusion (**Figures 6A,B**, *P* < 0.05).

## Discussion

Our study demonstrates that the neuroprotective effects of XNST are associated with its ability to: (1) attenuate BCCA ligation-induced demyelination, (2) reduce abnormal microglial activation, (3) facilitate microglia/macrophage polarization toward the M2 phenotype, resulting in restored neuronal plasticity and improved cognitive function, 42 days after chronic cerebral hypoperfusion in rats.

Cerebral circulation disturbances have been associated with a decline in cognitive function. BCCA ligation induces chronic cerebral hypoperfusion, resulting in a significant reduction of cerebral blood flow and impairment to memory and cognitive function. Previous studies have demonstrated that XNST provides neuroprotective effects by inhibiting apoptosis and inducing angiogenesis during acute cerebral ischemia, which is closely associated with memory (Zhang et al., 2008; Qian-Song et al., 2015). In this study, XNST reduced escape latency duration and increased platform crossing frequency. The present study demonstrates that treatment with XNST may mitigate memory impairments following chronic cerebral hypoperfusion.

Previous studies have demonstrated that signal formation and transmission play a key role in neurological function (Bouet et al., 2007), and that neuronal plasticity is closely associated with depression and memory (Busceti et al., 2015; Murphy, 2015). Many studies have shown that neuronal plasticity plays a vital role during ischemic injury, which impacts gray matter, white matter and synaptophysin (Castren and Hen, 2013; Pinheiro Fernandes et al., 2014; Murphy, 2015). Gray matter serves as an information processor and mainly contains neuronal cell bodies and unmyelinated axons. White matter plays an essential role in signal transmission and communication between different brain regions, and is mainly composed of bundles of myelinated axons, myelin-producing oligodendrocytes, and other glial cells. Myelinated axons play a key role in signal transmission and communication between different brain regions, however, the exposed nerve fiber is highly susceptible to degeneration and is linked to functional deterioration. Synaptophysin participates in regulating activity-dependent synapse formation (Yang et al., 2015). We found that the expression of MAP2 and synaptophysin were affected by BCCA ligation. This finding is in agreement with previous studies (Tarsa and Goda, 2002; Feng et al., 2012; Luo et al., 2016). In the present study, XNST attenuated disordered MAP2 expression, which was induced by chronic cerebral hypoperfusion. Meanwhile, XNST treatment improved synaptophysin levels. Myelin basic protein (MBP), a myelin marker, was assessed in this study. XNST significantly increased the MBP immunostaining intensity and protein expression, suggesting the remyelination of myelinated axons following chronic cerebral hypoperfusion. These findings suggest that XNST regulates neuronal plasticity following BCCA ligated-induced injury in gray and white matter regions.

Microglia cells play an important role in neuronal plasticity, such as, modulating synaptogenesis, synapse survival, and neuronal activity (Reemst et al., 2016). In addition, microglia also support neurogenesis in hippocampus by phagocytosing apoptotic cells (Reemst et al., 2016). However, excessive activation of microglia can lead to memory and cognitive function impairments following different types of brain injuries (Reemst et al., 2016; Sominsky et al., 2018). Recent studies have extended the therapeutic approaches should shift from broad suppression of microglia toward subtle adjustment of their phenotypes (Hu et al., 2015). Numerous studies have indicated that microglial phenotypic switching mediates white matter repair and axonal remyelination following cerebral injury (Hu et al., 2015; Wang et al., 2015). The pro-inflammatory M1 phenotype favors the production and release of cytokines that exacerbate neuronal injury. In contrast, the M2 phenotype promotes the release of neurotrophic factors that promote neuronal repair. In our current study, the M1 markers, CD16 and iNOS increased following BCCA ligation, but significantly decreased following XNST treatment. Protein levels of the M2 marker, Arg1, were further increased following XNST treatment. Consistent with our findings, previous reports have demonstrated that microglia/macrophage polarization is altered in other cerebrovascular diseases (Wang et al., 2013b; Li et al., 2015, 2016). Furthermore, previous studies have confirmed that microglia/macrophage polarization is associated with the AKT and ERK signaling pathways (Wang et al., 2015, 2016; Moretti et al., 2016). Consistent with these findings, our results demonstrate that BCCA ligation and XNST treatment enhances the expression of p-AKT (Thr308) and p-ERK (Thr202/Tyr204), which likely affects the phenotypic polarization of microglia/macrophages. Meanwhile, our results suggest that future anti-inflammatory therapies for cerebrovascular disease should focus on a more specific titration of the inflammatory response away from the destructive M1 phenotype.

In summary, this study demonstrates the neuroprotective effects of XNST treatment following chronic cerebral hypoperfusion in rats. XNST significantly ameliorates cognitive deficits via the: (1) restoration of neuronal plasticity, (2) reduction in demyelination, (3) prevention of polarization of the M2 to M1 phenotypes, and maintenance of the M2 microglia/macrophage phenotype, 42 days after BCCA ligation. This study provides novel insights into XNST treatment for chronic cerebral hypoperfusion.

## Author Contributions

LW contributed to experimental studies and manuscript preparation. RW aided in data acquisition. HZ helped in statistical analysis. ZC and YL concentrated on the concept, design, and manuscript revising. All authors read and approved the final manuscript.

## Conflict of Interest Statement

The authors declare that the research was conducted in the absence of any commercial or financial relationships that could be construed as a potential conflict of interest.
